# Advances in Folic Acid Biosensors and Their Significance in Maternal, Perinatal, and Paediatric Preventive Medicine

**DOI:** 10.3390/bios13100912

**Published:** 2023-09-28

**Authors:** Yogesh Movendane, Mbozu G. Sipalo, Leon C. Z. Chan

**Affiliations:** 1 Singapore Institute of Manufacturing Technology (SIMTech), Agency for Science, Technology and Research (A*STAR), 2 Fusionopolis Way, Innovis #08-04, Singapore 138634, Singapore; y.movendane@u.nus.edu; 2 Department of Biomedical Engineering, College of Design and Engineering, National University of Singapore, Singapore 117583, Singapore; 3 London School of Hygiene and Tropical Medicine, Keppel Street, London WC1E 7HT, UK; mbozu.sipalo@lshtm.ac.uk

**Keywords:** folic acid, biosensors, pre-eclampsia, cancer, point-of-care and perinatal healthcare

## Abstract

Auxotrophic primates like human beings rely on exogenous dietary vitamin B_9_ supplementation to meet their metabolic demands. Folates play a crucial role in nucleotide synthesis and DNA methylation. Maternal folate deficiency causes several pregnancy-related complications, perinatal defects, and early childhood cognitive impairments. New evidence suggests excess FA is a potential risk factor resulting in unfavourable genomic and epigenomic alterations. Thus, it is essential to revisit the need to consistently monitor maternal folate levels during pregnancy. Yet, to date, no point-of-care folate-monitoring biosensor is commercially available. Here, we critically appraise the advances in folate biosensors to understand the translational gaps in biosensor design. Further, our review sheds light on the potential role of folate biosensors in strengthening maternal, perinatal, and child healthcare.

## 1. Introduction

**Figure 1:**
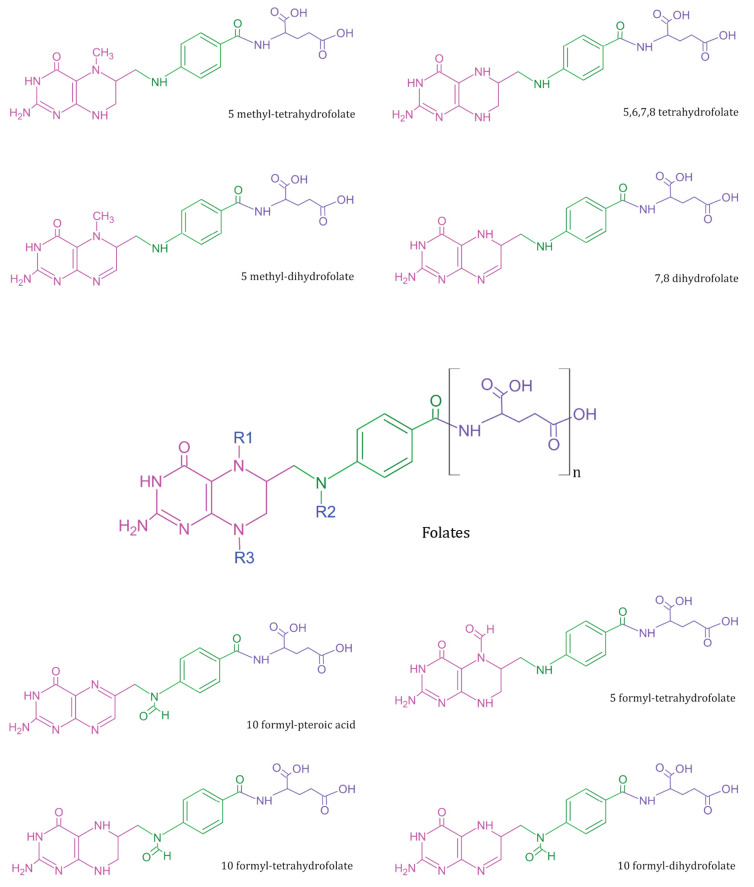
Summary of common folate vitamers—Folates (in centre) represent a general template structure, composed of a pteridine ring (in pink), p-aminobenzoic acid (in green), and glutamic acid moiety (in purple). Most metabolically active B_9_ vitamers exist in monoglutamate form and vary in structure based on R1, R2, and R3 substitutions.

Deoxyribonucleic acid (DNA), the genetic biopolymer repository synthesised and conserved within eukaryotic cells, is composed of deoxynucleotide triphosphate (dNTP) monomers. Deoxythymidine triphosphate (dTTP), a key dNTP, is synthesised by thymidylate synthase in the presence of folate derivative as cofactor. During pregnancy, the exponential increase in nucleotide biosynthesis supports rapid cell proliferation and tissue formation in the developing foetus. It creates a high metabolic demand for essential nutrients, usually quenched through maternal dietary intake or supplementation. Vitamin B_9_ or M (IUPAC name: (2S)-2-[[4-[(2-amino-4-oxo-3H-pteridin-6-yl)methylamino]benzoyl]amino]pentanedioic acid) is essential for nucleotide biosynthesis, DNA methylation, and amino acid homeostasis in cells [1]. Folates refer to a class of B_9_ vitamers with a basic structure composed of heterocyclic pteridine moiety covalently linked via a C_9_-N_10_ methylene bridge to p-aminobenzoylglutamate (Figure 1) [2]. The biological activity and bioavailability of B_9_ vitamers vary with oxidation state, substitutions, and the number of glutamate chains. Since humans lack the molecular machinery for de novo folate cofactor synthesis, the exogenous ingestion of natural folate polyglutamates and synthetic folic acid (FA) vitamer supplements support the nutritional demand. The ingested folate polyglutamates hydrolysed to monoglutamates, are converted to 5-methyltetrahydrofolate in the intestinal mucosa prior to entering circulation [3]. This circulated folate is then converted to 7,8-dihydrofolate (DHF) and 5,6,7,8-tetrahydrofolate (THF) by the cells to meet the metabolic demand [4,5]. 

The stability of B_9_ vitamers is reviewed elsewhere [6]. The normal range of folates is about 5–15 ng/mL in serum, 16–21 ng/mL in cerebrospinal fluid, and 175–316 ng/mL in erythrocytes [7]. To maintain the normal folate range, the daily recommended folate intake is 400 µg folate per day for adults [8]. Serum folate levels below the normal range indicate a deficiency condition. Multiple lines of evidence implicate folate deficiency in a variety of conditions during pregnancy that severely impact maternal and child health. Among such conditions, neural tube defects, pre-eclampsia, pre-term birth, stillbirth, spontaneous abortion, cognitive impairment, childhood cancers, polycystic ovary syndrome, and postpartum maternal mental health contribute substantially to the global health burden [9,10,11,12].

Among pregnant women who received at least 400 µg FA daily through fortified food and vitamin supplementations, complications such as neural tube defects dropped nearly by 50%. In women with a history of neural tube defects (high risk pregnancies), the daily dose of FA is recommended 1–3 months prior to conception [13]. Based on such well-substantiated evidence, strong folic acid fortification frameworks and policies are taking shape to tackle folate deficiency globally [14,15,16]. On the other hand, recent evidence suggests excess FA intake could also be detrimental to health [17]. 

Together, the danger of folate deficiency and excess highlights the need to monitor appropriate FA intake, especially during preconception and perinatal periods. At present, maternal folate levels are measured in clinical or laboratory settings. Intriguingly, an increase in stillbirth and neonatal mortality rates was observed across different countries during the COVID-19 pandemic [18,19]. This observation highlights the impact of dependency on clinical infrastructure for perinatal healthcare. Although unexpected, the need for point-of-care devices to bring quality perinatal healthcare to communities was evident during the pandemic. 

However, to date, no commercially available quantitative point-of-care folate biosensors exist. To understand the advances and challenges in folate biosensor research, we review their potential applications in maternal and child healthcare and critically appraise the existing FA biosensors. 

## 2. Folic Acid in Maternal, Perinatal, and Paediatric Health

Folates have been implicated in maternal and perinatal health for at least three decades. The role of various folates in metabolic homeostasis are summarised elsewhere [20,21,22]. In the following section, our focus is on the associations between maternal folate deficiency and different perinatal complications. 

### 2.1. Neural Tube Defects

The World Health Organization (WHO) reports an annual global estimate of 240,000 neonatal deaths within the first 28 days due to congenital disorders as of 2023. Neural tube defects (NTDs) occurring in weeks 3 or 4 of pregnancy are among the most common and extremely severe but preventable congenital disorders [23]. Anencephaly, the most fatal of NTDs, is a congenital brain malformation, where the anterior (cranial) neural arch fails to close during embryo development. This condition invariably results in stillbirth or neonatal mortality [11,24]. 

While anencephaly occurs early in embryogenesis, the same malformation occurring late in embryogenesis results in encephalocele. Typical brain-tissue protrusion through the skull, mostly in the occipital region, is a characteristic of encephalocele. It is relatively rare compared to anencephaly, yet presents a high mortality risk [25]. Another common NTD, spina bifida (SB), is a congenital spinal cord malformation, wherein the posterior (caudal) vertebral arch fails to close, resulting in meningocele, myelomeningocele, and/or hydrocephalus [11,26]. In the case of SB, though the mortality rate is relatively lower than anencephaly, the patients are likely to experience neurological disabilities. 

Within the proposed multifactorial aetiology of NTDs, nutritional deficiency arguably is the most easily preventable factor [27,28]. FA supplements and fortification were reported to reduce NTDs related to neonatal mortality [11,28,29,30]. In particular, two exemplary large-population, data-based analyses concluded periconceptual maternal FA intake prevents NTD occurrences [31,32]. Another meta-analysis of NTDs in eastern Africa also suggests that mandatory FA supplements could reduce the risk of NTDs [33]. Fascinatingly, an 11-year follow-up study by Caffrey et al. shows that continued FA supplementation post the timeframe recommended to prevent NTDs supports neurocognitive development in the baby [34].

### 2.2. Hypertensive Disorders

Pregnancy-induced hypertension (PIH) reduces nutrition and oxygen supply to the foetus. PIH includes chronic hypertension, pre-eclampsia (PE), superimposed pre-eclampsia, and gestational hypertension [35]. PE clinically manifests as hypertension and proteinuria occurring post-week 20 of gestation [36]. The aetiology of PE remains largely unknown. Moreover, unlike other pregnancy-related complications, PE is detrimental to both maternal and foetal health [37]. Strikingly, a key observation in PE and gestational hypertension is elevated levels of homocysteine in blood [12]. It is well-established that FA supplements mitigate the risk posed by elevated homocysteine levels [38]. 

Not surprisingly, controversial evidence for nutritional supplementation in reducing PE risk does exist [39,40]. Yet, the overwhelming amount of evidence highlights FA supplementation as a protective factor against PE [12,36,41,42,43,44]. 

### 2.3. Pre-Term Birth 

The WHO estimated around 13.4 million pre-term births (PTB) in 2020, which is a leading cause of neonatal mortality. For human beings, 40 weeks marks the normal gestation period. Any spontaneous pre-term labour between the 28th and 37th week of gestation is termed PTB [45]. The complications associated with PTB are diverse and include cerebral palsy, hypothermia, hypoglycaemia, brain injury, and cognitive impairment [46]. Tackling PTB is unquestionably a global priority. In May 2023, the United Nations International Children’s Emergency Fund (UNICEF) published an updated report on PTB, titled “Born Too Soon: Decade of action on preterm birth”. This report found that preterm births have not changed significantly in any region of the world in the past decade. Southern Asia and Sub-Saharan Africa account for more than 65% of preterm births recorded globally. In Singapore, nearly 9% of pregnancies end in PTB, and the trend is expected to increase in the near future [47]. 

Furthermore, this report proposes an action plan and identifies intersectoral risk factors influencing PTB, among which maternal nutrition is of high significance. Notably, a retrospective cohort study including 200,000 women observed a reduced PTB risk with FA supplementation [46]. Another case-controlled study reported low maternal serum folate levels in PTB cases [45]. 

A recent meta-analysis of epidemiological studies correlating PTB risk and maternal folate levels also conclusively states that PTB risk reduces with higher maternal folate levels [48]. Lastly, an observational study observing the high maternal folate level association with low PTB risk even proposed possible maternal folate level optimisation in the post-fortification era [49].

### 2.4. Stillbirth and Spontaneous Abortion

In 2014, 194 countries endorsed the “Every Newborn Action Plan (ENAP)” developed by the WHO. The ENAP sets a global target to achieve less than 12 stillbirths per 1000 total births in all 194 countries by 2030. Stillbirth is the loss of the foetus post week 20 of gestation. Approximately 1.9 million babies, or one every 16 s were born stillborn in 2021. The major causes of stillbirths include placental malperfusion, foetal asphyxia, congenital malformations, infection, PTB, PE, and umbilical cord complications [50,51,52]. Spontaneous abortion (SA) refers to the loss of a foetus prior to week 20 of gestation [53]. Several factors such as chromosomal abnormalities, nutritional deficiency, immunogenic factors, DNA fragmentations, lifestyle choices, and other factors could result in SA [53,54]. In accordance with such findings, studies suggest periconceptional FA supplementation reduces the risk of SA and stillbirths [55,56,57,58,59].

### 2.5. Early Childhood Cognition 

Cognition, occasionally dubbed the ultimate brain function, develops extensively during early childhood. Based on accumulating evidence highlighting the role of periconceptional FA supplementation in foetal neurodevelopment, studies hypothesised its influence on cognition and motor abilities. Fascinatingly, a study of children aged 4–5 years reported that low maternal FA levels resulted in attentional dysfunction preferentially among boys [60]. A longitudinal study of children aged 7–9 years, from the same group, reported that low maternal FA results in low alertness irrespective of gender. 

Interestingly, they also observed comparatively better cognitive and working memory among girls [61]. Another study conducted in Japan reports a higher cognitive, language, and social development quotient among 4-years-olds whose mothers began FA supplementation before week 12 of gestation [8]. Although such strong correlations are well-substantiated, certain previous studies providing insignificant correlations do exist [62]. This might be due to several postnatal factors influencing infant development through early childhood. 

### 2.6. Genomic and Epigenomic Instability

Most intriguingly, the latest sets of evidence hint at the need to study the optimal threshold for FA intake. As summarised above, low FA intake results in adverse pregnancy outcomes. On the other hand, excess FA supplement consumption could induce genomic and epigenomic instability [63,64]. Although further studies on the human population is essential to estimate the true impact of high and low FA intake, it is highly likely that excess FA intake could also result in unfavourable outcomes. 

### 2.7. Gestational Diabetes Mellitus 

Among different pregnancy-related complications, the most common metabolomic disorder is gestational diabetes mellitus (GDM) [65]. GDM refers to glucose intolerance occurring in early pregnancy and is estimated to affect 30% of pregnancies globally [66]. In Singapore, GDM is the most prevalent metabolomic pregnancy-related disorder, observed in roughly 25% of pregnancies. Several studies have reported a strong association between GDM and high FA with vitamin B12 insufficiency [67,68,69]. Although the aetiology of GDM remains elusive, current observations consistently report the imbalance in FA and vitamin B_12_ concentrations, postulating a potential role for surplus FA in GDM. 

## 3. Advances in Folate Biosensor Research

The analytical measurement of folates is continuously evolving, with the earliest gold standard being the microbial assay. A platform shift from microbial assay to chemical assay led to the development of the folate radioassay (Quantaphase) by Biorad laboratories. Obviously, due to safety concerns, these kits are no longer available. However, the discontinuation of radioassays created a space for folate-binding protein (FBP) assays, which to date remains the most preferred choice in clinical laboratories, apart from High Performance Liquid Chromatography (HPLC), Gas Chromatography (GC), and Mass Spectrometry (MS) techniques [3]. FBP assays are relatively economical compared to HPLC or MS techniques; however, they cannot distinguish between various vitamer forms. The current gold standards are thus largely limited to clinical and research laboratories equipped with appropriate analytical instruments.

Biosensors represent a class of analytical devices designed to precisely capture and transduce a signal, representative of a measurand or an analyte, into a qualitative or quantitative output. We recommend a detailed summary on biosensors available elsewhere [70]. Point-of-care (POC) devices in healthcare specifically refer to biosensors with an intended application, usable in non-clinical settings, with minimal to zero training or supervision. The WHO guidelines on an ideal biosensor outline affordable, sensitive, specific, user-friendly, rapid and robust, equipment-free, and deliverable to end-users (ASSURED) as the ideal characteristics or requirements [71]. Advances in microfabrication and manufacturing techniques have enabled the commercial availability of such POC applications with near-ideal characteristics. The notable commercial success of a POC could be considered to reflect end-user demand and preferences. 

**Figure 2:**
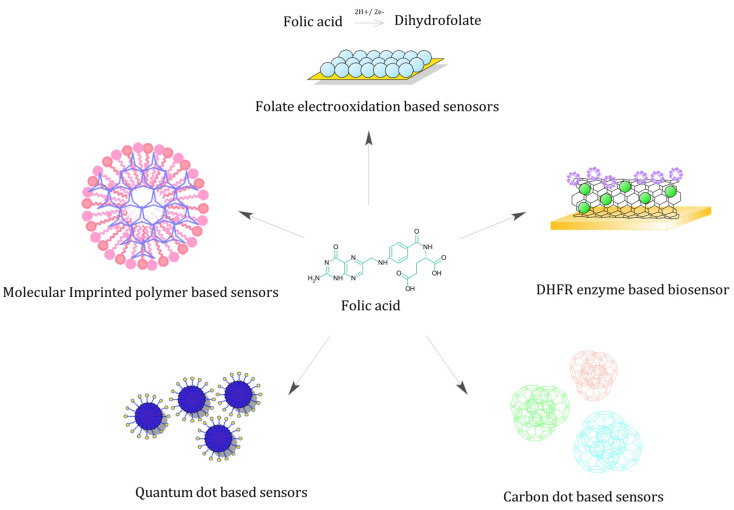
Folate biosensors—Schematic representing various folate biosensors based on unique sensing strategy.

The previous section summarised the association between maternal folate levels and major pregnancy-related complications. In the following section, we intend to comprehensively review the existing folate biosensors and their merits. Remarkably, we found nearly all folate biosensors to exclusively fall under electrochemical or optical biosensor categories (Figure 2).

### 3.1. Electrochemical Biosensors

Electrochemical biosensors are perhaps the most common sensing strategy adopted irrespective of the analyte, given it can selectively elicit an electrical signal. In electrochemical biosensors, key performance factors such as sensitivity, specificity, stability, and precision greatly influence the sensing element design. Widely known for their design simplicity, robustness, and scalability, they remain a popular choice among transduction systems used in biosensor design. As expected, we found an overwhelming amount of electrochemical folate biosensors.

#### 3.1.1. Sensing Strategies 

Early reports on pretreated carbon fibre microelectrodes designed to determine FA from synthetic FA stock solution yielded a detection limit of 10^−8^ M and a linear range of 2 × 10^−8^ M to 10^−6^ M. The peak current response elicited at pH 1 in a Britton–Robinson (BR) buffer and 0.1 M perchloric acid was suggested for analytical use [72]. Such highly acidic working pH is not the best fit for an optimised clinical assay. At physiological pH 7.4, a solid gold electrode fabricated with a self-assembled monolayer of 2-mercaptobenzothiazole achieved a detection limit of 4 × 10^−9^ M and a linear range of 8 × 10^−9^ M to 10^−6^ M, within 120 s accumulation time using a stock solution of pharmaceutical FA dissolved in 0.1 M NaOH and PBS (Phosphate-Buffered Saline) buffer [73].

Similar to the previous work, a gold electrode coated with multi-walled carbon nanotube (MWCNT) yielded a detection limit of 10^−8^ M and a linear range of 2 × 10^−8^ M to 10^−6^ M within 420 s accumulation time using similar pharmaceutical formulation [74]. However, this MWCNT electrode elicited a peak response at pH 2.5 and interference from vitamin B_2_ and haemoglobin, limiting its application. It is noteworthy that electrode surface area and electroactive target diffusion influence the sensor performance, and thus, minimizing interference is of the utmost significance. 

Besides gold electrodes, other materials such as lead films, mercury, carbon paste electrodes, and electrodes fabricated with nanoparticles were also investigated. Lead film electrodes were proposed as an alternative to toxic mercury electrodes used in adsorptive stripping voltammetry. Lead film electrodes exhibited a detection limit of 7 × 10^−10^ M and a linear range of 2 × 10^−9^ M to 5 × 10^−8^ M for pharmaceutical FA samples dissolved in NaOH, adjusted to pH 5.6 using an acetate buffer with an accumulation time of 300 s [75]. Though lead is relatively less toxic than mercury, a preference towards non-toxic materials exists. Calixarenes exhibit relatively low toxicity. 

A p-tert-butyl-calix(6)arene coated graphite powder used in the fabrication of a carbon paste electrode (CME6) yielded a detection limit of 1.24 × 10^−12^ M and a linear range of 8.79 × 10^−12^ M to 1.93 × 10^−9^ M [76]. The optimal electrode response was observed at pH 4.0 adjusted with BR buffer and an accumulation time of 120 s. It is also noted that ascorbic acid interferes with FA detection by CME6. Due to C_9_-N_10_ oxidation in FA generating the signal, and due to electrochemical inactivity in glutamic acid within the studied potential window, these electrodes usually lack the ability to distinguish between monoglutamates and polyglutamates. 

All the above studies demonstrated the ability to detect FA in artificial formulations. The following studies summarised below predominantly focus on novel sensing materials to achieve better performance in real-time samples such as pharmaceutical tablets, food samples, serum, blood, and urine.

1. Folates in serum samples

As early as 2009, Palraj et al., reported an ultrathin 5-amino-2-mercapto-1,3,4-thiadiazole modified GCE with an optimal pH of 7.2, exhibiting a detection limit of 0.23 × 10^−9^ M and a linear range of 10^−7^ M to 0.8 × 10^−3^ M. This electrode was highly selective to FA even in the presence of ascorbic acid and uric acid and performed well in human serum samples with a relative standard deviation (RSD) of 1.24% [77]. Another GCE modified with a composite of phosphomolybdic acid-polypyrrole/graphite yields a detection limit of 3.3 × 10^−11^ M with a linear range of 10^−9^ M to 2 × 10^−7^ M using serum samples with an RSD of 1.5% [78]. A similar GCE fabricated with MoS_2_/reduced graphene oxide composite yielded a detection limit of 10^−8^ M and linear range of 10^−8^ M to 10^−4^ M, with an RSD of 6.3% [79]. Though these electrodes achieved low detection limits and optimal working conditions suitable for serum samples, they lacked long-term stability. The electrodes exhibited a current response loss of 5.23% [77], 7% [78], and 4.36% [80] after 2 weeks, seriously limiting their use.

In addition to modified GCE, a gold electrode fabricated with α-polyoxometalate-polypyrrole-Au nanoparticles yielded a detection limit of 0.12 × 10^−9^ M at pH 6.0 adjusted with a BR buffer and an approximate RSD of 5% [80]. An interesting nanocomposite composed of a zeolite-like metal organic framework doped with silver nanowires fabricated on a screen-printed carbon electrode reported a detection limit of 30 × 10^−9^ M with a linear range of 10^−7^ M to 10^−5^ M and an RSD of 3.06% [81]. 

Unlike the above-mentioned tests, wherein folate detection is restricted to the electrode surface area, molecularly imprinted polymers sense folates in a three-dimensional cavity complementary to the folate structure. A pencil graphite electrode modified with a hyperbranched polymer made of 2,4,6-trisacrylamido-1,3,5-triazine, a trifunctional monomer, exhibited a detection limit of 0.0021 µgmL^−1^ and linear range of 0.007 µgmL^−1^ to 0.156 µgmL^−1^ (RSD −3%) with an accumulation time of 120 s [82]. However, the optimal working pH was found to be 2.5 since both the FA and molecularly imprinted polymer are relatively neutral and exhibit strong hydrophobic interaction. 

**Figure 3:**
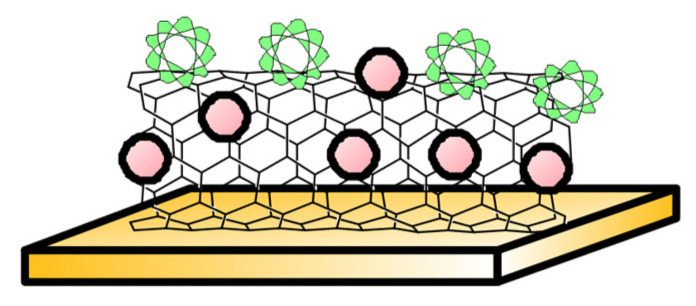
Enzyme-based electrode—Sketch depicts Dihydrofolate reductase (in green) immobilised to multiwalled carbon nanotubes and TiO_2_ nanoparticles (in red) on solid gold electrode, reported in [83].

Lastly, while substantial research focuses on folate oxidation-based electrochemical sensing, they are not selective to a specific vitamer structure. Such vitamer specificity was achieved using a gold electrode fabricated with multi-walled carbon nanotubes, titanium dioxide nanoparticles, and a dihydrofolate reductase enzyme (Figure 3). This experiment reported a detection limit of 11.48 nM and linearity over 5 nM to 50 nM with a sensitivity of 0.42 µA/nM/cm^2^ at pH 7.5 [83]. The sensor yielded a standard deviation of about 3.9% between serum sample batches collected from pregnant women. Owing to the DHFR enzyme, the sensor is selective to 7,8 dihydrofolate and the sensor stability was found to decrease by 45% in 5 months.

2. Folates in urine samples

The exceptional efficiency of carbon nanotubes (CNT) in heterogenous catalysis conferred by their high surface area, electrical conductivity, and material strength remain undisputed. However, the production of uniform and economical carbon nanotubes is challenging. Ordered mesoporous carbon (OMC) was proposed as a green chemistry alternative with similar efficiency, with an added advantage of relatively easier production with tuneable uniform pore size. A GCE coated with OMC as the transducing layer enabled an extremely low detection limit at 6 × 10^−11^ M with linearity over 5 × 10^−10^ M to 10^−7^ M at pH 7 (adjusted with BR buffer) and an accumulation time of 10 s [84]. This electrode yielded an RSD of 2.66%.

Similarly, room temperature ionic liquids (RTIL) have also been explored as a green alternative to conventional electrode materials. A carbon ionic liquid paste with infused ZnO nanoparticles-based electrode was reported to exhibit a detection limit of 10^−8^ M with linearity over 5 × 10^−8^ M to 1.5 × 10^−6^ M (sensitivity 1.776 µA/µM) and 1.5 × 10^−6^ M to 550 × 10^−6^ M (sensitivity 0.033 µA/µM) at pH 9 (adjusted with PBS) with 2.1% RSD [85].

Due to ease of biomolecule adsorption and relative stability of ZnO nanoparticles at physiological pH, it is often a preferred component in sensor fabrication. Still, ZnO by itself has relatively poor electrical conductivity and requires an efficient conductive layer to act as sensitive electrochemical sensor material. One such electrode design is the graphene-ZnO nanoarrays with graphene foam. This electrode achieved a detection limit of 1 µM with linearity over 1 µM to 60 µM (sensitivity 0.18 µA/µM) at pH 7.4 (adjusted with 0.01 M PBS) [86]. With similar desirable attributes to ZnO nanoparticles, α-Fe_2_O_3_ nanoparticle modified GCE designed to simultaneously detect uric acid, folic acid and riboflavin was reported. This electrode yielded a detection limit of 25 nM with linearity over 0.11 µM to 691 µM at pH 7.4 (adjusted with PBS) and an RSD of 2.6% for FA [87]. 

It is noteworthy that, at all instances, the urine samples tested were diluted with a working buffer by a dilution factor of 5 to 10. While urine folate measurement provides a user-friendly option compared to serum folate measurement, clinically relevant factors such as creatinine correction, history of chronic kidney disease, hydration status and so on must be accounted for in translation.

3. Folates in food samples

Natural dietary folates exist in both monoglutamate and polyglutamate forms. However, folic acid supplementations almost exclusively contain monoglutamate vitamers because of their high bioavailability. Several electrodes have measured folates from food samples by folate redox reaction using electrochemical approaches. With high electrical conductivity, chemical and mechanical stability, inert electrochemical activity over a broad potential window, and relative affordability, GCE is perhaps the most common electrode modified to detect folates. 

As previously discussed, both carbon nanotubes and room temperature ionic liquids possess highly desirable qualities for electrode fabrication. Fei et al. investigated the performance advantage of CNT–RTIL combined electrodes. They reported a single-walled carbon nanotubes (SWCNT) modified GCE with 1-octyl-3-methylimidazoliumhexafluorophosphate as the binding component, achieving a detection limit of 10^−9^ M and linearity over 2 nM to 4 µM at pH 5.5 (adjusted with PBS) and an accumulation time of 360 s [88]. The electrode performance was reproducible with 3.95% RSD. 

Similar sensor performance was achieved using nanoparticle modified GCEs. Recently, a MoS_2_ nanosheet modified GCE was reported to have a detection limit of 32 nM and linear range from 0.06 µM to 24.3 µM at pH 6 (adjusted with 0.1 M BR buffer) and an RSD of 4.71% [89]. Another GCE modified with Mo_2_C-sulphur doped graphitic carbon nitride yielded a detection limit of 14.7 nM with linearity over 0.09 µM to 167.25 µM at pH 7 (adjusted with PBS). The electrode exhibited an RSD of 3.52% and about 6.8% response loss over 4 weeks [90]. Lastly, a GCE modified with Cu(II)O nanoparticles and electropolymerised polymethyl orange realized a detection limit of 2 nM with linearity over 0.01 µM to 1.5 µM at pH 7 with an RSD of 0.37% and only 1.6% response loss over 50 days [91]. Like most electrochemical folate sensing electrodes, these electrodes sense folate vitamers irrespective of glutamate status. However, it is not a critical requirement for sensors designed to determine folate from food samples. 

An unconventional approach to folic acid determination, with a disposable pencil graphite modified with salmon dsDNA using response surface method, is worth a mention. This electrode reported a detection limit of 0.106 nM with linearity over 0.1 µM to 10 µM at pH 4.8 for 24 µg/mL DNA [92]. An RSD of 4.6% for 2 µM FA and response loss of 3.1% in 45 min was observed. 

The electrodes discussed above have demonstrated their ability to sense folates in complex biological matrices. A few other folate biosensors have also been reported to achieve excellent sensitivity and selectivity in artificially prepared pharmaceutical samples (Table 1).

#### 3.1.2. Challenges

With the extensive testing summarised above, it is clear that highly sensitive, specific, stable, and reproducible sensing strategies are available for electroanalytical folate detection. Yet, to date, nearly all such strategies are confined within research laboratories. This breakdown in translation could likely be due to technical and fabrication challenges morphing into stability, reproducibility, and sensitivity issues. 

In electrochemical biosensors, a common fabrication strategy is to form a monolayer in the case of CNTs or nanoparticles, or coatings in the case of thin film polymer layers. In both cases, the leaching or wear and tear of sensing material is a major concern. This material loss might often translate into loss of current response over time, accounting for standard deviations in electrode performance and fluctuations. In other words, the rate of sensing material deterioration is reflected in decreasing precision and stability.

Secondly, to be relevant in healthcare, the sensing element resilience enabling analyte specificity in complex biological samples such as serum, cerebrospinal fluid, or plasma matrix is essential. Issues such as biofouling, interference from structurally similar molecules, and non-optimal conditions are inevitable. Though a variety of anti-fouling strategies are available, implementing them requires additional electrode modification steps or a robust sample-preparation strategy. 

Thirdly, the mechanical and morphological characteristics of electrodes largely rely on fabrication process and operational conditions. For instance, stability is a key issue with boron doped diamond electrodes, while geometry control is a key issue in CNT fabrication. Likewise, the mechanical and conductive properties of ceramic electrodes are largely impacted by preparation processes. Hence, it is essential to meticulously evaluate materials for electrode fabrication based on the intended application during the design phase. 

Lastly, the robustness of signal-generating sensing elements must also be considered. Some folate biosensors using highly sensitive antibodies, enzymes, oligonucleotides, and other bioconjugates often require an appropriate sample pre-processing step. These biosensors must also be stored in specific conditions for optimal and reliable performance. Morever, the need for an electrical readout component often results in these biosensors falling short of ASSURED criteria. However, recent advances in low-cost fabrication with green chemistry principles, such as paper microfluidic biosensors, are successful to an extent in overcoming these issues. 

### 3.2. Optical Biosensors

Optical biosensors are relatively recent and offer an alternative detection technique to electroanalytical biosensors. These sensors are known for high selectivity, less laborious sample-preparation steps, and rapid real-time monitoring even in biologically complex samples. One of the early folate biosensors with commendable performance reported in 1991 was an electrochemical biosensor. However, the first optical folate biosensor was reported only in 2000. This optical folate biosensor employed an immunoaffinity-based sensing strategy. The study reported a precision varying between 88% and 101%, with a 4% to 10% reproducibility relative standard deviation [101]. Still, in the last two decades, less than 20 optical folate biosensors were reported (summarised below in Table 2).

#### Challenges

Optical biosensors are unique in design and thus present unique set of challenges as well. A frequent problem with fluorescence-based sensing strategies is improving signal strength. Quantum dots often employed in biosensors depend on the stability of the core shell. Similarly, a fluorescence-quenching strategy involves a meticulous sensing matrix fabrication, which is both laborious and sensitive to environmental conditions. However, the most important challenge among optical biosensors is the effective capture of the generated signal. Although optical biosensors result in a spectral shift in response to the changes in analyte concentrations, it is difficult to reliably interpret such shifts with the naked eye. Hence, some kind of recording set-up is often required. In certain instances, like lateral flow assays or colourimetric paper microfluidic biosensors, wherein an obvious colour change yields a semi-quantitative or merely qualitative result, such readout set-ups may not be essential. In most cases, the need for quantitative readout options is still required. While some studies focus on developing smartphone-based readout applications, the possible variations in focal length and camera resolution remains a challenge. Overall, the development of robust signal capture strategies is an active area of research that could likely resolve major challenges enabling the translation of optical biosensors.

## 4. Discussion

Arguably, biosensors offer several advantages in terms of portability, affordability, and time without compromising on sensitivity, specificity, and reliability, compared to standard analytical techniques such as HPLC or GC-MS. At present, the prevalent use of FBP assays is restricted to clinical settings. Many excellent and well-designed studies with substantial cohort size have established the positive correlations between folate deficiency and several disease conditions. Such disease conditions range from cardiovascular diseases to cognition, cancer, congenital malformations, pre-eclampsia, pre-term birth, stillbirth, and other pregnancy-related complications. Furthermore, there is accumulating evidence suggesting the possible detrimental impact of a FA surplus, including genomic and epigenomic instability. Correspondingly, several guidelines exist on recommended folate dosage, but a global consensus on precise optimal folate levels during various stages of pregnancy is required. The current clinical practice to prevent folate deficiency during pregnancy is to prescribe excess folate supplements, since folates do not exhibit acute toxicity. However, patient compliance remains a concern. Moreover, as folate tests exist as laboratory-oriented procedures, access to them in low-resource settings, as well as during unforeseen circumstances like times of pandemic, is limited. 

The prevalence of neonatal mortality due to NTDs still has not been eradicated. A significant population of pregnant mothers face the risk of pre-eclampsia, preterm births, hypertensive disorder-related complications, chronic kidney disease, gestational diabetes mellitus, and stillbirths, especially in low-resource settings. In accordance with policies and intersectoral action plans published by the WHO and UNICEF to combat infant mortality and morbidity risks, multi-pronged research approaches are being developed to strengthen the quality of perinatal healthcare.

Despite decades of advances in electrode design to measure folic acid, no POC (at-home) folate biosensors are yet available. This suggests several unbridged gaps in the translation and commercialisation of folate biosensors. For instance, some folate biosensors measuring serum folate levels were summarised earlier. As previously highlighted, challenges such as biofouling, interference, and limited specificity hinder the clinical usability of these biosensors. However, a more serious gap is the lack of integration of the designed electrodes into an actual prototype. Biosensors with specifically designed sample preparation strategies, or microfluidic components integrated with the electrodes, were found to be extremely limited in number. Resolving these gaps could likely expedite the realisation of an affordable, portable, reliable in vitro point-of-care device for early folic acid deficiency. It is imperative since, although excess folate supplementation has shown no signs of acute toxicity, it has not eradicated folate deficiency. Ultimately, non-invasive, continuous folate monitoring or economical disposable POCT biosensors would prove to be an invaluable tool in screening for nutritional deficiency during pregnancy.

Another limiting factor is the support required in collating large-cohort data to explore the true impact of folate deficiency in disease conditions. Although a few large-cohort studies with substantial sample size were reported in recent years, the correlation trends observed must be re-evaluated regularly. This is important because, with some countries focussing on FA fortification programmes, there is a possibility of variations in the trends reported based on the effectiveness of such fortification programmes. It can be argued that such variations highlight the economic disparity among different countries. Regardless, such long-term trend-monitoring would yield data to analyse the impact of healthcare accessibility on the management of the folate deficiency-related disease burden. The commercial translation of such screening tools could potentially reduce the global healthcare burden by strengthening maternal and child healthcare. We conclude this review with a call for an interdisciplinary approach to realise POCT folate biosensor translation. 

## 5. Future Directions

Through this review, we shed light on the potential impact of folate biosensors on maternal and child healthcare. Further, we have also highlighted a few critical challenges involved in the design of folate biosensors. Despite such efforts spanning the past three decades, a portable POCT folate biosensor remains unrealised. As summarised in this paper, folate levels play a crucial role during pregnancy and must be frequently monitored. Hence, an affordable, sensitive, real-time monitoring folate biosensor with a short response time could be an invaluable tool, preventing infant mortality, disability, and other healthcare burdens related to pregnancy. In the future, we plan to realise a POCT folate biosensor and evaluate its applicability in maternal and perinatal healthcare.

## Figures and Tables

**Table 1:** Performance characteristics of electrochemical folate biosensors recorded in pharmaceutical samples.

Sensing Electrode	Detection Limit	Linear Range	pH	Time (s)	Recovery (%)	RSD (%)	References
Cu-SnO_2_ modified GCE	0.024 nM	0.1 nM to 67 µM	7.0	5	98.3 to 100.1	2.73	[93]
Bismuth nanowire modified GCE	9.53 nM	10 nM to 150 nM	4.5	240	90 to 94	2.5	[94]
Hydroxyapatite modified GCE	75 nM	0.1 µM to 0.35 mM	7.0	5	98.48 to 103.25	5.6	[95]
Graphite-Polymethacrylic acid MIP-SiO_2_	1.63 nM	0.01 µM to 0.23 µM	4.5	-	97.7 to 105	5.01	[96]
BSA modified gold nanoclusters	18.3 ng/mL	120 ng/mLto 33.12 µg/mL	7.4	300	93.5 to 95.7	1.49	[97]
Boron doped diamond electrode	0.32 µM	2.3 µM to 90 µM	6.0	120	95.1 to 101.4	2.6	[98]
Interwoven Ti nanotubes and carbon nanohorns	0.025 nM	0.1 nM to 50 µM	7.0	-	96.1 to 99	11.8	[99]
ZrO_2_ modified carbon paste electrode	9.86 µM	20 µM to 2.5 mM	7.0	5	98.85 to 103.55	3.1	[100]

**Table 2:** A summary of different sensing strategies employed in optical folate biosensors.

Method	Sample	Sensing Mechanism	Detection limit	Linear Range	RSD (%)	References
Electrochemiluminescence	Urine	Electrooxidation of FA in presence of NaNO_3_ as supporting electrolyte	10^−8^ gmL^−1^	10^−7^ to 10^−5^ gmL^−1^	5.5	[102]
Chemiluminescence	Pharmaceutical sample	FA reacts with Ru(bipy)32+ and Ce (IV)	23 nM	0.31 µM to 25 µM	3.5	[103]
	Pharmaceutical and urine samples	FA reacts with diperiodatoargentate (III) in presence of Cu nanoclusters	69.8 nM	0.1 µM to 10 µM	1.36	[104]
Immunochromatographic Assay	Milk powder sample	Immunogen FA-BSA and coating antigen FA-OVA prepared by a carbodiimide-modified active ester method.	51.8 ngmL^−1^	23.4 to 114.5 ngmL^−1^	16.7	[105]
Fluorescence	Pharmaceutical samples	Fluorescence quenching of Eu-based metal organic framework by FA	0.12 mM	1 mM to 9 mM	4.05	[106]
Quantum Dots	Pharmaceutical samples	Fluorescence quenching of MoS_2_ QD by FA	0.1 µM	0.1 µM to 125 µM	2.8	[107]
Spectral correlation interferometry	Artificial FA samples	FA antigen—antibody interaction	0.9 pM	0.9 pM to 220,000 pM	-	[108]
Carbon Dots	Fortified food and pharmaceutical samples	Carbon dots functionalised on cellulose by Schiff’s base chemistry	0.28 µM	1 µM to 300 µM	4.5	[109]
	Pharmaceutical samples (artificial serum)	Carbon dots synthesised with anhydrous citric acid precursor and ethylenediamine	6 nM	0 nM to 50 nM	10.4	[110]

## Data Availability

Not available.
